# Comprehensive Structural Characterization of Wheat Bran Lignin

**DOI:** 10.1021/acs.jafc.4c11880

**Published:** 2025-04-05

**Authors:** Gijs van Erven, Romy J. Veersma, Mirjam A. Kabel

**Affiliations:** a Laboratory of Food Chemistry, Wageningen University & Research, Bornse Weilanden 9, Wageningen 6708 WG, The Netherlands; b Wageningen Food and Biobased Research, Bornse Weilanden 9, Wageningen 6708 WG, The Netherlands

**Keywords:** wheat bran, lignin, pyrolysis-GC-MS, HSQC NMR, lignocellulose fractionation

## Abstract

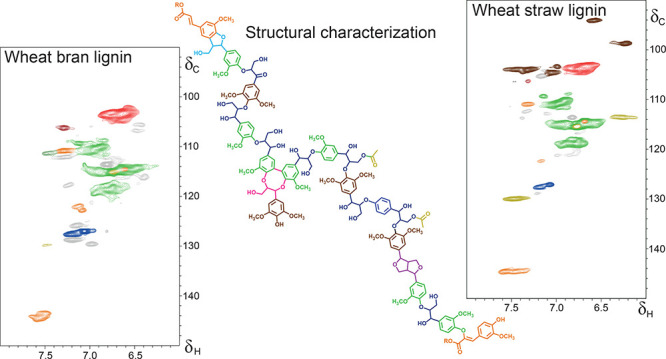

Wheat bran is a large volume sidestream of wheat flour production and is used in food and feed applications. Despite great advances in the characterization of the wheat bran hemicellulose component, thus far, only limited attention has been paid to wheat bran lignin. Here, we describe the comprehensive structural characterization of wheat bran lignin, facilitated by sequential enzymatic starch and protein removal, followed by mild γ-valerolactone organosolv extraction and extensive purification. Quantitative ^13^C-IS pyrolysis-GC-MS and HSQC NMR revealed that wheat bran lignin is enriched in syringyl subunits (S/G ∼ 0.9), as compared to wheat straw lignin (typical S/G ∼ 0.5), but surprisingly poor in *p*-coumarate incorporated (<1 per 100 aromatic rings), entirely free of tricin, and accordingly composed of typical β-O-4 aryl ether (84%), β-5 phenylcoumaran (7%), and β–β resinol (9%) interunit linkages. Moreover, and in line with the interunit linkage abundance (46 per 100 aromatic rings), alkaline SEC and ^31^P NMR, respectively, confirmed a macromolecular nature (*M*_w_ 6900 g/mol, *Đ* 5.9) and low phenolic hydroxyl content (1.6 mmol/g of lignin) of the wheat bran lignin structure. Our extensive characterization efforts contribute to the dedicated valorization of wheat bran lignin and support understanding potential physiological effects when incorporated into human and animal diets.

## Introduction

Wheat (*Triticum* spp.) is cultivated as main human food crop in the European Union.^1^ The wheat kernel is composed of germ, starch-rich endosperm, and bran cell wall tissues, constituting 2–3%, 80–85%, and 13–17% of the grain dry matter, respectively.^1^ Wheat bran is obtained as byproduct of the wheat milling and flour refinery processes and mainly utilized in bakery, cereal foods, and animal feeds.^2^

Industrial wheat bran is typically composed of endospermic starch (20–30%), proteins (∼15%), the cell wall polysaccharides cellulose (10–15%) and hemicellulose (∼25%) and lower amounts (∼5%) of fats, minerals, and lignin.^3,4^ Being the main fiber component, extensive research has been devoted to wheat bran hemicellulose, primarily comprised of glucuronoarabionoxylan (GAX). GAX consists of a β-(1→4)-linked xylopyranosyl backbone that is substituted by α-l-arabinofuranosyl units at the *O*-3 or *O*-3 and *O*-2 position, and by (4-*O*-methyl-)α-d-glucuronosyl units at the *O*-2 position.^5^ The arabinosyl units can be esterified at their *O*-5 position to ferulic or diferulic acid and, although in much lesser extents, to *p*-coumaric acid.^6^ The ferulate substructures are involved in intramolecular cross-linking of GAX as well as etherification to lignin (GAX-FA-Lignin).^7,8^

In contrast to the well-studied GAX structures, the literature on the wheat bran lignin structure is scarce. Nonetheless, the structure of wheat straw lignin has been extensively investigated.^9,10^ As such, it is well-known that lignin in wheat straw and, in other grasses, is typically composed of *p*-hydroxyphenyl (H), guaiacyl (G), and syringyl (S) subunits that are linked through various aryl ether and carbon–carbon linkages, with the β-*O*-4’ aryl ether as most abundant interunit linkage.^11^ In grasses, these interunit linkages are partially acylated at the γ–OH position with acetate and *p*-coumarate.^12,13^ Recently, wheat straw lignin was shown to incorporate substantial levels of the flavonoid tricin, further adding to the complexity of its structure.^9^

Still, the presence of “true” lignin in wheat bran has been confirmed by selective cleavage of lignin β-*O*-4 bonds and mass spectrometric detection of the released degradation products.^14,15^ Presumably, the main reason behind the lack of structural information on wheat bran lignin relates to its previously shown challenging isolation when following traditional protocols, giving extremely low yields (<10 mg/g insoluble fiber) combined with an insufficient purity for detailed characterization.^16^ Furthermore, any structural information implied from lignin characterization after severe pretreatment and/or fractionation^17^ should be treated with caution at the very least, if not avoided, for the known impact of such treatment conditions on the lignin structure. Recently, mild lignin isolation from maize bran was more successful, furthermore showing unique lignin structures incorporating diferuloylputrescine.^18^ This raises the question of whether such structural features could also be present in other cereal brans, such as wheat bran.

Given the considerable volumes of wheat bran consumed, both by humans and animals,^19^ we argue that it is important to better understand how wheat bran lignin contributes to the physiological effects of the fiber component on gut microbiota, feed passage, and digestion. Importantly, health benefits of wheat bran consumption, such as antioxidant capacity coupled to prevention against colon cancer, have largely been ascribed to its ferulic acid constituents, but lignin might be involved as well.^20^ Therefore, we consider it relevant to characterize the wheat bran lignin structure in detail.

Hence, in this work, mild organosolv lignin fractionation, proven to well preserve the native lignin structure,^21,22^ was applied to obtain a representative lignin extract from enzymatically destarched and deproteinated wheat bran, and followed by extensive purification and comprehensive structural characterization by state-of-the art lignin analytics. By comparison to a wheat straw lignin preparation, important structural features of wheat bran lignin are highlighted.

## Experimental Section

### Materials

All chemicals used were purchased from Sigma-Aldrich (St. Louis, MO, USA), Merck KGaA (Darmstadt, Germany) or VWR International B.V. (Amsterdam, The Netherlands). The water used in all experiments was purified with a Milli-Q water system (Millipore, Billerica, MA, USA). Wheat bran was supplied by Koopmans Meel BV (Leeuwarden,The Netherlands). Wheat straw was obtained from Royal Agrifirm Group (Apeldoorn, The Netherlands) and, prior to analyses, milled through a 1 mm sieve at 12,000 rpm (ZM200; Retsch GmbH, Haan, Germany). The cellulase-enriched commercial enzyme cocktail was purchased from Sigma-Aldrich, the xylanase enriched commercial enzyme cocktail Viscostar 150L was obtained from Dyadic (Jupiter, FL, USA) and the xylanase-enriched commercial enzyme cocktail Rovabio Advance was purchased from Adisseo (Antony, France)

### Acetone Extraction

Three portions of wheat bran (175 g) were each mixed with 1.6 L of acetone and extracted at 40 °C for 2 h with manual shaking at 15 min intervals. Insoluble residues were obtained through filtration using a 40 μm pore size polypropylene membrane (Sefar AG, Thal, Switzerland) in a headspace overpressure device with max 2 bar air pressure. Insoluble residues were combined and subsequently air-dried prior to starch and protein removal.

### Starch and Protein Removal

Acetone extractive-free wheat bran was milled through a 0.5 mm sieve at 10,000 rpm (ZM200; Retsch). Next, milled wheat bran was dispersed in water (8% w/w) and dosed with 125 μL of thermophilic α-amylase (Megazyme Ltd., Wicklow, Ireland) per g starch (starch content 10% w/w)^23^ and incubated at 100 °C in a water bath for 1 h with manual agitation every 15 min. Another 375 μL of α-amylase was then dosed, and incubation was continued for another hour (with intermittent shaking every 15 min). Bottles were subsequently cooled on ice to reach 30–40 °C and dosed with protease from *Bacillus lichteniformis* (type VIII; Sigma-Aldrich) at 3.2 mg protease per g protein (protein content 16% w/w).^23^ Sodium azide was added to reach a final concentration of 0.01% w/w to prevent microbial growth, and bottles were incubated at 55 °C in a shaking incubator for 18 h. Insoluble residues were obtained through filtration using the same setup as described above, washed twice with 1 L of water, and freeze-dried, prior to its further use for dioxane or mild organosolv lignin extraction.

### Dioxane Lignin Extraction

Freeze-dried destarched-deproteinated wheat bran was bead-milled as described above, and further planetary ball milled using a PM100 planetary ball mill (Retsch, Haan, Germany) as published.^24^ Two portions of ball-milled material (9 g each) were mixed with 100 mL of 50 mM sodium acetate buffer (pH 5.0) and dosed with the three commercial (hemi)cellulolytic enzyme cocktails Cellulysin (2.5% of dry matter (dm)), Rovabio Advance (1% of dm), and Viscostar 150L (0.5% of dm). Additional buffer was then added to obtain 5% w/w sample concentration, followed by a 48 h incubation in an oven with head over tail rotation (20 rpm) at 40 °C. After incubation, samples were centrifuged (2500*g*, 20 °C, 5 min) and washed three times with 100 mL of Milli-Q. Insoluble residues were then mixed with dioxane to reach 80% v/v aqueous dioxane and a 10% w/w dm concentration. Samples were continuously purged with nitrogen gas flow, covered with aluminum foil, and magnetically stirred at 750 rpm at room temperature. After 24 h, the samples were centrifuged (4700*g*, 10 min, 20 °C), and insoluble residues were extracted with 50 mL of 80% v/v aqueous dioxane each and extracted for a second 24 h cycle as above. Supernatants of both extractions were combined, frozen at −20 °C, and freeze-dried to obtain the crude wheat bran dioxane lignin extract.

### Mild Organosolv Wheat Bran Lignin Extraction

Mild γ-valerolactone (GVL) fractionation conditions were based on previous reports established to well preserve the lignin structure.^21,22^ Hereto, freeze-dried destarched-deproteinated wheat bran (150 g) was mixed with 1.5 L of solvent (80% w/w GVL, 19% w/w water, 1% w/w sulfuric acid) in a 2 L stainless steel reactor vessel and treated in a 4524 Parr reactor connected to a 4848 reactor controller (Parr Instrument Company, Moline, IL, USA). After closing the reactor, stirring was set at 2.5 (a.u.) and the target temperature was set at 120 °C. The internal reactor temperature reached the target temperature in 30 min, and the reaction was continued for another 30 min. The reaction was stopped by rapidly cooling the reactor to 60 °C with running tap water in 5 min. Reactor contents were separated by centrifugation (15,000*g*, 10 min, 20 °C). The insoluble residue was washed with 500 mL of fresh reaction solvent, and soluble dark liquor fractions were combined. Lignin was precipitated from the dark liquor by addition to 8 times the volume of water and allowed to fully settle at 4 °C for 4 days. Precipitated lignin was separated by centrifugation (15,000*g*, 10 min, 20 °C), washed three times with 200 mL of water that was set at pH 2 and air-dried to yield 7.3 g of crude wheat bran lignin.

### Mild Organosolv Wheat Straw Lignin Extraction

Small-scale mild organosolv fractionation was performed according to van Erven et al. (2023).^22^ Hereto, 8 mL of solvent (80% w/w γ-valerolactone, 19% w/w water, and 1% w/w H_2_SO_4_) was added to 0.5 g of wheat straw material. After thoroughly vortexing, samples were added to a Stuart SBH200D/3 heating block (Cole Palmer, Vernon Hills, IL, USA), set at 120 °C. After extraction (45 min, vortexing every 5 min), the samples were cooled on ice and centrifuged (2,500*g*, 2 min, 20 °C). Next, 6 mL of supernatant was transferred to 40 mL water, vigorously mixed, and left to precipitate (24 h, 4 °C). Precipitated lignin was obtained by centrifugation (4,700*g*, 2 min, 20 °C), washed twice with 25 mL of water (set to pH 2), and air-dried at room temperature to obtain crude wheat straw organosolv lignin.

### Wheat Bran Lignin Purification

A portion of 2.5 g crude wheat bran lignin was mixed with 25 mL petroleum ether (40–60 °C) (PE), thoroughly vortexed, and sonicated for 30 min. The insoluble residue was obtained through centrifugation (2,500*g*, 2 min, 20 °C), washed with another 25 mL of PE, and dried at 40 °C under a stream of nitrogen.

Three portions of PE-extracted lignin (500 mg each) were added to glass reaction tubes and extracted through mild dioxane acidolysis as reported.^25^ Hereto, 10 mL of 0.25 M HCl in 90% v/v aqueous dioxane was added, and samples vortexed thoroughly and added to a Stuart SBH200D/3 heating block (Cole Palmer, Vernon Hills, IL, USA) set at 100 °C for 1 h with brief vortexing every 10 min. After the treatment, the tubes were cooled on ice and centrifuged (2500*g*, 2 min, 20 °C), supernatants were removed, and residues were washed with 5 mL of 0.25 M HCl in 90% v/v aqueous dioxane. All supernatants were combined, added to 300 mL of water to precipitate the lignin, and allowed to settle at 4 °C for 24 h. The precipitated lignin was obtained through centrifugation (4700*g*, 5 min, 20 °C), washed twice with 20 mL of water (pH 2), and freeze-dried. Next, 700 mg of the resulting mild acidolysis lignin was mixed with 14 mL chloroform/ethanol 50:50 v/v, vortexed, sonicated for 20 min, and centrifuged (2500*g*, 2 min, 20 °C) to separate the close to completely solubilized material. The insoluble residue was washed with 5 mL of chloroform/ethanol and centrifuged, and the supernatant was added to the soluble fraction. Lignin was precipitated by addition to 8 times the volume of petroleum ether and allowed to settle at 4 °C for 1 h. The precipitated lignin was obtained through centrifugation (2500*g*, 2 min, 20 °C), washed with 40 mL petroleum ether, and dried at 40 °C under a stream of nitrogen to yield approximately 0.6 g of purified wheat bran lignin.

### Wheat Bran Compositional Analysis

Dry matter, ash, fat, protein, starch, and nonstarch polysaccharide contents were determined in (untreated) wheat bran as described previously, all following standardized and validated protocols.^26^ The Klason lignin contents of the wheat bran, destarched and deproteinated wheat bran, and wheat bran lignin isolate were determined as published.^26^ The Klason lignin content is here defined as acid-insoluble lignin (AIL), corrected for residual ash, protein, plus acid-soluble lignin, where the destarched and deproteinated wheat bran and wheat bran lignin isolate AIL was additionally corrected for nonlignin apolar substances, as published.^26^

### Wheat Bran Hydroxycinnamic Acid Extraction and Analysis

The total (free and ester-bound) hydroxycinnamic acid (HCA) content in freeze-dried deproteinated and destarched wheat bran was determined according to published protocols,^27,28^ with some modifications. Prior to extraction, samples were milled to a particle size of ≤ 250 μm (MM400, Retsch). Hereto, 2 g of sample was milled in a 50 mL stainless-steel jar with two φ15 mm stainless-steel balls at a frequency of 30 Hz for 2 min. For extraction of free HCAs, 500 μL of 50 mM sodium acetate buffer (pH 5.7) was added to 10 mg of sample. For extraction of esterified HCAs, 500 μL of 0.5 M KOH was added to a 10 mg sample. All sample preparations were performed in duplicate, including two controls containing 50 μg mL^–1^ ferulic acid and *p*-coumaric acid to correct for HCA stability during sample preparation. Samples were incubated for 5 h at 37 °C with continuous mixingat 350 rpm in an Eppendorf incubator (Eppendorf Inc., Hamburg, Germany). For improved mixing, the incubator was placed at a 45° angle. Aluminum foil was used to cover the Eppendorf incubator and minimize light exposure to the samples. Next, samples were centrifuged (5000*g*, 10 min, 20 °C) and, subsequently, supernatants were acidified to pH 5 by addition of 7 M acetic acid. Acidified samples and controls were stored overnight at room temperature. When applicable, samples were diluted 10–100 times with water. Samples were analyzed on a Vanquish reverse phase-ultra high performance liquid chromatography (RP-UHPLC) coupled to photodiode array detection (PDA) and electrospray ionization (ESI) mass spectrometry (MS) (Thermo Scientific, Waltham, MA, USA), based on the method described by Lancheros et al. (2023).^28^ Samples (1 μL) were injected on an Acquity UPLC BEH C18 column (150 mm × 2.1 mm, 1.7 μm particle size) with a VanGuard guard column of the same material (Waters, Milford, MA, USA). Column and guard column temperatures were set to 45 and 40 °C, respectively, and autosampler temperature was kept at 10 °C. A flow rate of 400 μL was applied with water (A) and acetonitrile (B) eluents, both acidified with 0.1% formic acid. The elution profile was as follows: 0–2 min 5% B (isocratic), 2–15 min linear increase of 5–40% eluent B, 15–16 min linear increase of 40–100% eluent B, 16–20 min 100% eluent B (isocratic), 20–21 min linear decrease from 100 to 5% eluent B, and finally, 21–26 min 5% eluent B (isocratic). MS data was recorded in negative ionization mode at *m*/*z* 120–1500, and ferulic acid and *p*-coumaric acid [M–H]^−^ ions were detected at *m/*z 193 and *m/*z 163, respectively. Nitrogen was applied as a sheath and auxiliary gas. For identification of di- and triferulates, the MS/MS analysis was performed with collision-induced dissociation (CID) with a normalized collision energy of 35 eV. The transfer tube temperature was set at 263 °C, source heater temperature at 425 °C, and the source voltage at 2.5 kV. Quantification of ferulic acid and *p*-coumaric acid was based on 322 and 310 nm, respectively, and calibration curves (0.1–75 μg mL^–1^; *R*^2^ = 1.0) of the two HCAs, with peak integration in Genesis mode (FreeStyle 1.8 SP1, Thermo Scientific). The peak areas of *trans*- and *cis*-ferulic acid were combined for quantification of ferulic acid. The amount of ester-bound ferulic acid and *p*-coumaric acid content was determined as amount total HCAs minus amount free HCAs. According to Underlin et al. (2020),^27^ several diFAs and a triFA were quantified by PDA. Hereto, the FA calibration curve was used (area versus molarity) and assumed that the molar response factor of diFA and triFA was equal to the molar response of FA.

### Quantitative Pyrolysis-GC-MS with ^13^C Lignin as an Internal Standard

To quantify lignin content and structural features, lignin fractions were analyzed by quantitative pyrolysis-GC-MS. Analytical pyrolysis coupled to gas chromatography with high-resolution mass spectrometric detection (Exactive Orbitrap, Thermo Scientific) was performed as previously described, using an Agilent VF-1701 ms column (30 m x 0.25 i.d., 0.25 μm film) for chromatographic separation.^29^ The split ratio was 1:133 for the first 5 min and 1:13.3 for the remaining 52 min to reduce helium usage without compromising method performance. Uniformly, ^13^C-labeled lignin (97.7 atom % ^13^C), isolated from ^13^C wheat straw (Iso*Life* BV, Wageningen, The Netherlands) was used as an internal standard (^13^C-IS).^30^ To each accurately weighed sample (80 μg) was added 10 μL of ^13^C-IS solution (1 mg mL^–1^ ethanol/chloroform 50:50 v/v) and dried prior to analysis. All samples were prepared and analyzed in duplicate. Lignin-derived pyrolysis products were monitored in full MS mode on the most abundant fragment per compound (both nonlabeled and uniformly ^13^C-labeled). Pyrograms were processed by TraceFinder 4.0 software (Thermo Scientific). Lignin contents and relative abundances of lignin-derived pyrolysis products were calculated as described previously.^29^ Lignin contents were also conservatively calculated excluding *p*-hydroxyphenyl units and 4-vinylguaiacol to avoid potential interference by aromatic amino acids and ferulic acid, respectively.

### HSQC NMR

Approximately 20 mg of sample was dissolved in 0.6 mL of DMSO-*d*_6_ for NMR measurements. Measurements were performed on a Bruker AVANCE III 600 MHz NMR spectrometer (Bruker BioSpin, Rheinstetten, Germany) equipped with a 5 mm cryo-probe located at MAGNEFY (MAGNEtic resonance research FacilitY, Wageningen, The Netherlands). ^1^H–^13^C HSQC spectra were recorded by using the adiabatic “hsqcetgpsisp2.2” pulse sequence using the following parameters: spectral width of 7,200 Hz (12 ppm) in F1 (^1^H) using 4096 increments for an acquisition time (AQ) of 0.29 s, an interscan delay (D1) of 1.0 s, and a spectral width of 33,000 Hz (220 ppm) in F2 (^13^C) using 512 increments with an AQ of 8 ms with 16 scans per increment. The^1^*J*_CH_ used was 145 Hz. Processing used Gaussian apodization (GB = 0.001, LB = −0.2) in the ^1^H dimension and a squared cosine function (SSB = 2) in the ^13^C dimension. The central solvent peak was used as an internal reference (δ_C_ = 39.5 ppm; δ_H_ = 2.49 ppm). The spectra were processed using TopSpin 4.0 software (Bruker). Semiquantitative analysis of the HSQC volume integrals was performed according to del Río et al. (2012), making use of the chemical shifts reported in the literature for annotation.^9^

### ^31^P NMR

Approximately 30 mg of the purified lignin fraction was phosphitylated and analyzed as previously described.^31,32^

### Size-Exclusion Chromatography (SEC)

Alkaline SEC was performed according to Constant et al. (2016; Method D),^33^ with the purified lignin fraction dissolved in 0.5 M NaOH (eluent) at 1 mg mL^–1^ concentration and UV 280 nm detection as previously described.^32^

## Results and Discussion

### Wheat Bran Lignin Content Is Overestimated by Klason Methodology

The wheat bran used in this study showed a chemical composition as expected (Table S1) based on the literature.^34^ All measured constituents combined accounted for over 97% of the dry matter. Admittedly, the Klason lignin content observed (13.3% w/w) is presumably considerably overestimated, even after correcting AIL for ash and protein.^35^ The content of lignin in cereal grains has been a topic of debate because the standard gravimetric determinations after sulfuric acid hydrolysis lead to a severe overestimation through interference of acid-insoluble nonlignin components such as protein and lipids.^35^ Indeed, the destarched and deproteinated wheat bran already showed a substantially lower Klason lignin content (AIL corrected for protein, ash, and fat + ASL; Table S2), as was confirmed by ^13^C-IS pyrolysis-GC-MS (Table S2). It should be noted that a wheat straw lignin internal standard was used for the latter analysis; not ideal for quantifying the lignin content of the bran with a fundamentally different structure as disclosed herein and elaborated upon below.^29^ Still, the ^13^C-IS pyrolysis-GC-MS method allowed us to surely exclude any interference from nonlignin components and confirmed the presence of actual lignin (2.6% w/w; Table S2). To arrive at the latter conservative lignin content, all *p*-hydroxyphenyl pyrolysis products and 4-vinylguaiacol, known to majorly derive from aromatic amino acids and from ferulic acid, respectively, were excluded from the lignin content calculations.

### Wheat Bran Lignin Can Be Isolated with Decent Yields and Extensively Purified

**Figure 1:**
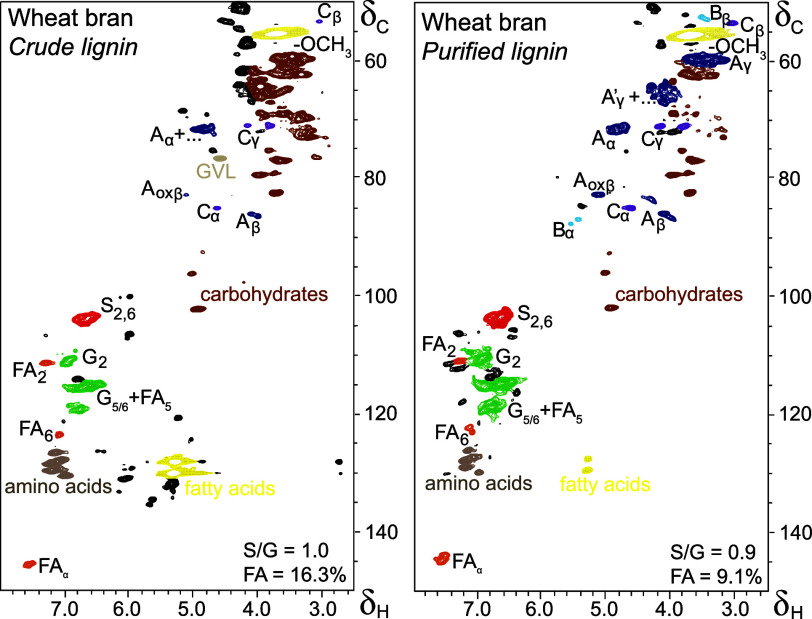
HSQC NMR spectra of crude and purified wheat bran lignin. Colors for the main lignin-related signals match Figure 2. Fatty acid, aromatic amino acids, carbohydrate, and residual γ-valerolactone extraction solvent impurities are respectively colored.

An initial attempt to isolate lignin from wheat bran using enzymatic carbohydrate removal followed by traditional dioxane extractions resulted in extremely low lignin yields (<5% of Klason lignin) and had insufficient purity (<20% w/w) to allow further analysis. HSQC NMR analysis of the dioxane extract confirmed the impurity of the sample (Figure S1). Only traces of lignin-derived signals could be discerned. Similar isolations from wheat straw gave 75% yield and 90% w/w purity.^36^ Even though such traditional protocols are consistently accepted to represent all lignin present for a scala of biomass feedstocks,^37^ apparently, to isolate lignin from wheat bran, another approach had to be applied. Recently, a mild gamma-valerolactone-based organosolv technology has been developed,^21^ proven to allow isolating lignin in high yields, while preserving the native structure from a variety of high lignin feedstocks such as poplar^38^ and miscanthus.^22^ Here, we applied this technology to wheat bran, seen as a low(er) lignin content feedstock for the first time. The mild organosolv lignin extraction of destarched-deproteinated wheat bran yielded 7.3 g crude lignin isolate, well exceeding the 4 g of lignin theoretically present in the 150 g starting material based on the conservative pyrolysis-GC-MS lignin content estimations (2.6% w/w; Table S2). This suggested coextraction and coprecipitation of nonlignin constituents. Indeed, HSQC NMR of the crude lignin isolate (Figure 1) showed overwhelming signals of fatty acids (vinyl signals δ_C_/δ_H_ 129.3/5.4; 127.5/5.3 ppm), besides signals attributed to aromatic amino acids (phenylalanine δ_C_/δ_H_ 128.8/7.2 ppm) carbohydrates (e.g., δ_C_/δ_H_ 69.1/3.3 and 72.8/3.2 ppm) and residual γ-valerolactone extraction solvent (δ_C_/δ_H_ 76.5/4.6 ppm). Given the promising yield of this first crude lignin isolate, we continued with extensive, yet mild, purification. Encouraged by a previously shown preservation of the lignin structure,^25^ in terms of subunit composition, but also in terms of interunit linkages, tricin and *p*-coumarates, we employed the same purification approach in this study. The effectiveness of the purification of the crude lignin isolate is here (Figure 1) highlighted by a complete removal of residual γ-valerolactone, 99% reduction of fatty acids, 68% reduction of phenylalanine, and near complete disappearance of some carbohydrate-derived peaks in the aliphatic region and that while preserving the lignin structure as suggested by the S/G ratio proxy (Figure 1). Given the overlap of nonlignin signals and likely interference in the crude lignin sample, we stress that detailed structural analysis of such impure samples should be performed with great care at the very least but may better be avoided.

Klason lignin analysis of the purified wheat bran lignin isolate corrected for any remaining ash, protein, and apolar interferences^26^ presented a lignin content of 82% w/w (76.2% w/w AIL + 5.7% w/w ASL). To the best of our knowledge, this has never been achieved for such a complex lignin source until now. The purified wheat bran lignin isolate was obtained in a yield of 42%. Satisfied with the lignin purity and yield, we subjected the sample to our comprehensive lignin toolkit for detailed structural analysis.

### Wheat Bran Lignin Is Syringyl-Rich, Poor in *p*-Coumarate and Free of Tricin

Pyrolysis-GC-MS showed a typical grass-like lignin fingerprint for all wheat bran fractions, with *p*-hydroxyphenyl, guaiacyl, and syringyl units all being present, next to the typical 4-vinylguaiacol and 4-vinylphenol products indicative of ferulate and *p*-coumarate, respectively (Table S3). Nonetheless, the relative abundance of lignin-derived pyrolysis products for wheat bran, in particular the purified wheat bran lignin, was rather different than we, and others, have thus far observed for various wheat straw, and wheat straw lignin isolate samples (Table S3).^9,29^ Compared to wheat straw (WS) lignin, the obviously enriched syringyl abundance of the wheat bran (WB) lignin stood out (tSinA/tConA WB = 1.66; WS = 0.55), besides higher levels of true H-units as derived from the abundance of *trans*-coumaryl alcohol (tCouA_WB_ = 5.9%; tCouA_WS_ = 3.8%) and substantially lower levels of 4-vinylphenol (4-VP_WB_ = 4.0%; 4-VP_WS_ = 12.5%). Though an increase in syringyl abundance can be recognized by pyrolysis-GC-MS during isolation and purification of the wheat bran lignin, we suggest that this relative increase was mostly caused by the reduction of (remaining) levels of ferulic acid as fractionation proceeded (indicated by 4-VG abundance). Indeed, crude and purified wheat bran lignin showed a comparable S/G when analyzed by HSQC NMR (Figure 1).

**Figure 2:**
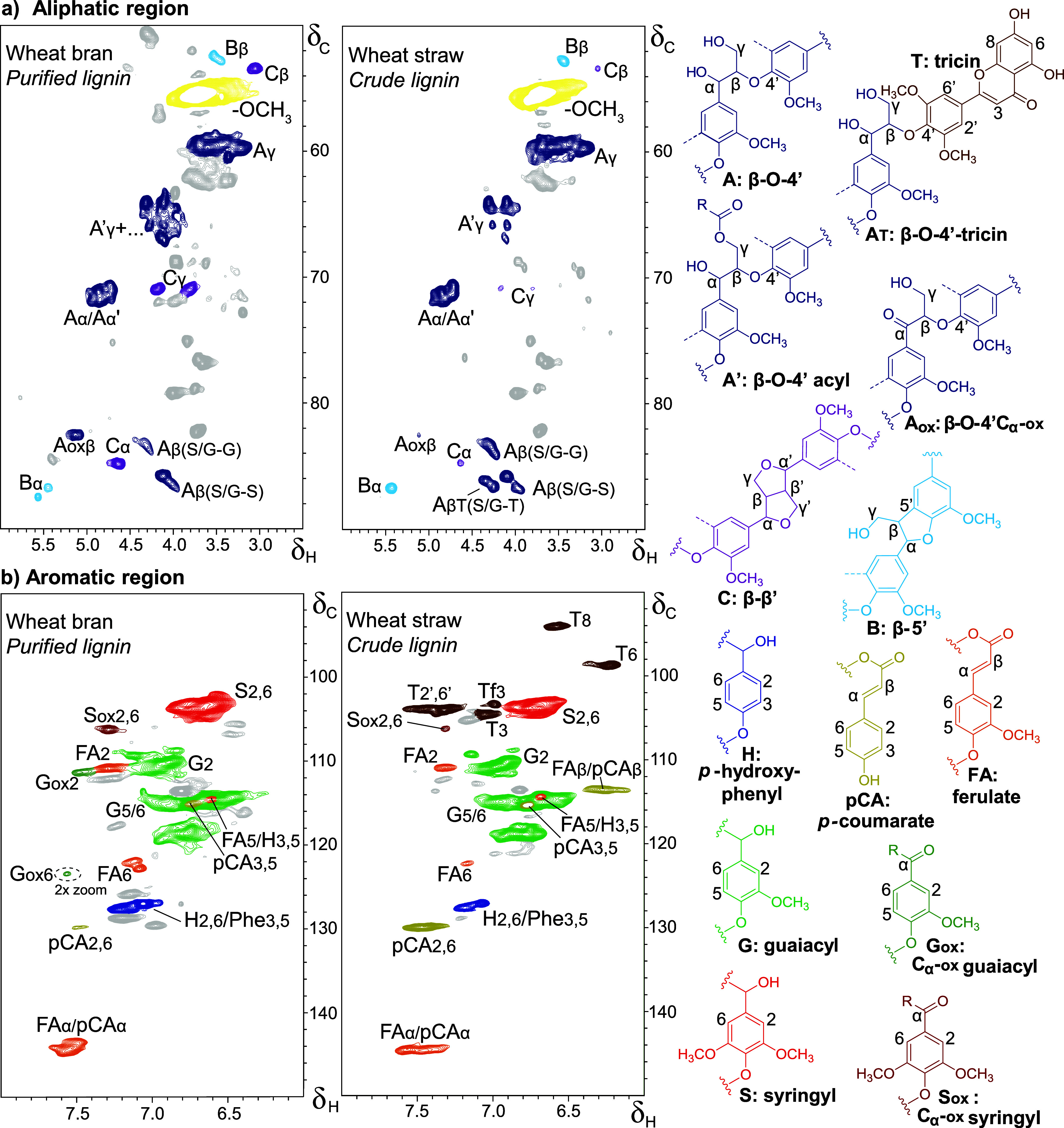
(A) Aliphatic and (B) aromatic regions of HSQC NMR spectra of purified wheat bran and crude wheat straw mild organosolv lignins. Unassigned peaks are shown in gray.

To further look into the structure of wheat bran lignin, the purified isolate was subjected to detailed HSQC NMR analysis and compared to a wheat straw lignin isolate that was obtained in a similar fashion. This wheat straw lignin preparation did not require extensive purification given the sufficient purity of the crude isolate obtained (Figure 2).

The crude wheat straw organosolv lignin used for comparative purposes here was structurally similar to a previously characterized wheat straw lignin preparation obtained through identical means and in turn was similar to native wheat straw lignin.^24^

NMR spectra (Figure 2) and semiquantitative analysis of the volume integrals (Table 1) pinpointed a clearly S-enriched lignin structure of wheat bran as compared to wheat straw (S/G_WB_, 0.89; S/G_WS_, 0.58).

**Table 1:** Semiquantitative HSQC NMR Analysis of Volume Integrals of Purified Wheat Bran and Crude Wheat Straw Mild Organosolv Lignin Isolates

	**wheat bran**	**wheat straw**
subunit composition (molar %)		
H	0.2	2.9
G	49.0	61.0
G_ox_	3.7	0.4
S	44.2	34.7
S_ox_	2.9	1.0
S/G	0.89	0.58
interunit linkages (per 100 ar)^a^		
β-O-4 aryl ether	32.0 (69.9)	45.6 (88.3)
β-O-4 aryl ether Cα-ox	6.4 (14.1)	0.3 (0.6)
β-5 phenylcoumaran	3.3 (7.3)	5.2 (10.0)
β-β resinol	4.0 (8.8)	0.6 (1.1)
total	45.8 (100)	51.7 (100)
end units (per 100 ar)		
cinnamaldehyde	0.5	1.6
hydroxycinnamic acids (per 100 ar)		
ferulate	9.7	5.7
*p*-coumarate	0.8	14.3
flavonoids (per 100 Ar)		
tricin	0.0	20.3

a Relative abundance of interunit linkage in parentheses; ar: aromatic rings.

Moreover, the analysis surfaced a 20 times lower abundance of p-CA moieties in wheat bran (0.8 per 100 aromatic rings) compared to wheat straw (14.3 per 100 aromatic rings) (Table 1). The abundance of H-units in HSQC NMR was likely skewed due to the substantial overlap of phenylalanine units, despite our purification attempts and correction of the volume integrals through well-resolved signals.^39^ This once more emphasizes the need for improved methods for detecting true H-units in any type of lignin samples. Relative coumaryl alcohol quantification through pyrolysis-GC-MS might help out in that respect (Table S3).

Intriguingly, the wheat bran lignin spectra further surfaced the complete absence of tricin, while wheat straw is known to be one of the most tricin-rich lignins described thus far,^40^ and shown here once more with tricin being quantified at 20.3 per 100 aromatic rings (Table S3). It has been proposed that tricin could function as a “nucleation site” for lignin formation, as alternative to the resinol structures that are presumed to start a lignin chain after monolignol dimerization.^41,42^ The presence of tricin therefore has been considered to underly the low abundance of resinol structures in typical grass lignins.^41,42^ Following this line of thought, indeed, wheat bran lignin showed a clear, relatively high resinol content of 4.0 per 100 aromatic rings (Table 1). In fact, the relative abundance of resinol structures was 8 times higher compared to wheat straw lignin and close to the levels found for typical hardwood lignins.^43^ Ferulic acid substructures have been ascribed an analogous nucleation role as tricin,^8^ and they were more abundantly present in the wheat bran lignin as well (Table 1). This could be related to the reported higher feruloylation degrees of the wheat bran cell wall. Importantly, saponification of the wheat bran lignin isolate showed that only a small portion of the ferulate and diferulates present could be released (data not shown), confirming that they were, in fact, present in etherified form to the lignin. Per definition, these substructures should therefore be considered (and quantified) as lignin. Also, this piece of evidence makes it unlikely that said ferulates were present as monolignol conjugates, that is, esterified at the Cγ-position. Interestingly, a closer look at the wheat bran lignin HSQC spectrum (Figure 2B) showed abundant signals corresponding to FA_α_ (δ_C_/δ_H_ 144.5/7.5 ppm), while signals of the typical FA_β_ partner (δ_C_/δ_H_ 113.5/6.3 ppm) were absent. We therefore infer that ferulic acid was either primarily present in the form of β–β (or 8–8′)-linked diferulates or that (di)ferulate structures were bound to lignin via the β position.

Besides the resinol structures, the wheat bran lignin further consisted of typical β-*O*-4 aryl ether and β-5 phenylcoumaran interunit linkages (Figure 2; Table 1). The substantial abundance of Cα-oxidized β-*O*-4 aryl ethers (6.4 per 100 aromatic rings) was partially due the syringyl-enriched structure of wheat bran lignin, being inherently more prone to oxidation, though Cα-oxidized guaiacyl motifs were also present. This oxidation likely occurred during the isolation and purification, as indicated by the increase in Cα-oxidized pyrolysis products as fractionation proceeded (Table S3) and thus should be mainly viewed as slight workup artifacts.

In grass lignins, β-*O*-4 aryl ethers are typically found to be acylated at the γ position.^13^ Despite the near absence of *p*-coumarates in wheat bran lignin, signals for acylated β-*O*-4 aryl ethers could be readily observed (Figure 2), thus implying that these units are likely acetylated instead. We deliberately opted out of semiquantifying their abundance based on the HSQC spectra, because the signals might (still) be overlapped with carbohydrate-derived peaks, even though the purification steps undertaken significantly reduced carbohydrate interference.

**Figure 3:**
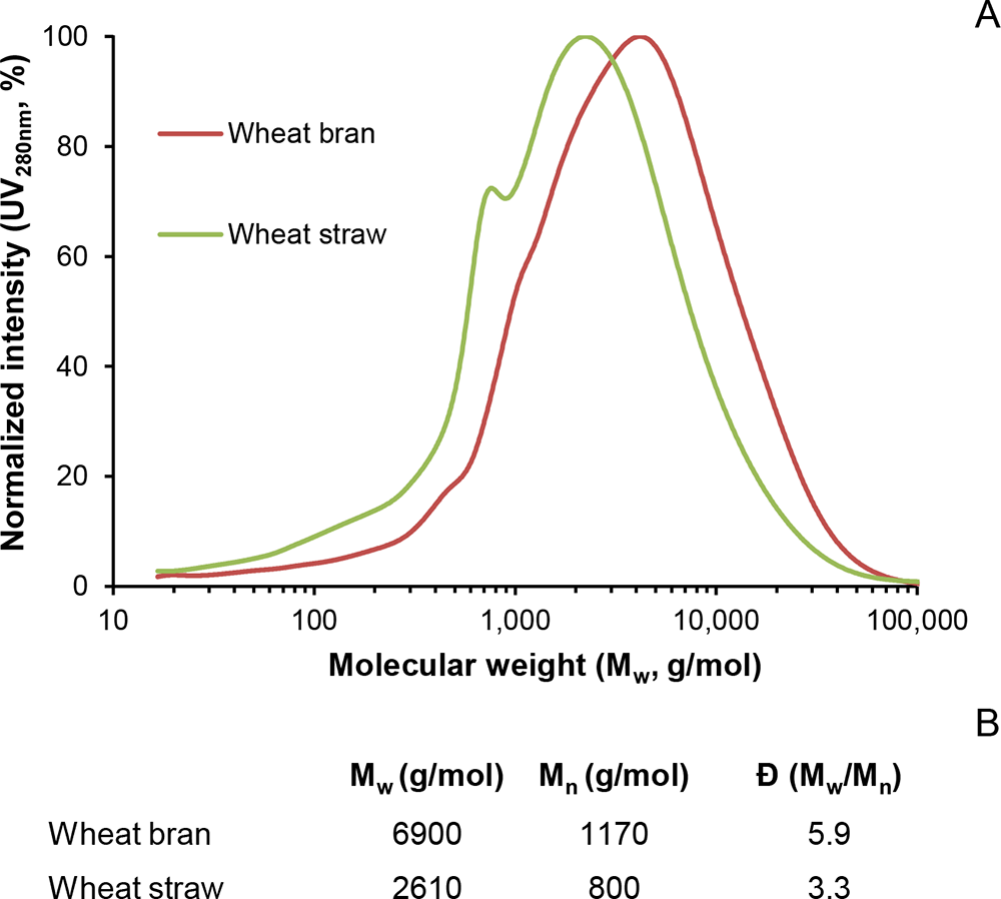
(A) Alkaline SEC elution profiles and (B) molecular weight distribution of purified wheat bran lignin and crude wheat straw mild organosolv lignins.

The subunit composition of the wheat bran lignin was also reflected in ^31^P NMR spectra, showing that phenolic syringyl contents exceeded that of the guaiacyl and *p*-hydroxyphenyl counterparts (Table S4). The overall low phenolic content of the wheat bran lignin determined (1.6 mmol/g lignin) is furthermore in line with the highly conserved interunit linkage content (Figure 2; Table 1) and this is also reflected in the macromolecular nature and high molecular weight observed in SEC analysis (Figure 3). Note that the molecular weight of the wheat bran lignin isolate (*M*_w_ = 6900 g/mol) was determined to be considerably larger than that of the wheat straw lignin preparation (*M*_w_ = 2610 g/mol). Given our extensive purification efforts and similar UV response and similar areas under the curve in the SEC analysis (data not shown), we consider it unlikely that the observed difference in molecular weight is solely due to covalently attached (nonlignin) cell wall constituents. Wheat bran lignin might in fact be truly larger than wheat straw lignin. Combined with the complex matrix wheat bran lignin is embedded in, at low contents, this could explain the difficulties encountered during its isolation when following traditional protocols.

In line with the negligible release of hydroxycinnamic acids through saponification, alkaline SEC analysis of the wheat bran lignin (Figure 3) neither showed the presence of monomeric/dimeric structures, in stark contrast to the wheat straw lignin reported here, and that of highly *p*-coumaroylated miscanthus lignins published previously.^22^

Interestingly, the above observations that wheat bran lignin is syringyl-enriched, reduced in *p*-coumarate, and free of tricin, as compared to wheat straw lignin all match well with the recent characterization of the lignin structure of maize bran,^18^ and comparison of that lignin to maize straw lignin analogues. However, the structural similarity was not extended to the unique diferuloylputrescine incorporating lignin structures of the maize bran demonstrated.^18^ Our spectra hold no indications for the presence of diferuloylputrescine substructures in wheat bran lignins.

In conclusion, we have demonstrated that the structure of lignin in wheat bran is considerably different than the structure of wheat straw lignin in terms of subunits, interunit linkages, hydroxycinnamic acids, and flavonoids incorporated. Showing that such large structural differences in the lignin polymer can already be found within parts of the same plant implies that there is a vast amount of lignin structures still to be characterized, a challenge and opportunity at the same time. From another viewpoint, our work provides a cautionary note for referring to lignin structures on the basis of their phylogeny alone. Though large-scale industrial batches of wheat bran and wheat straw were used here, further research will need to establish the effect of among other plant variety, growth stage, and cultivation conditions on the lignin structures present. Being syringyl rich, we argue that the contribution of lignin to wheat bran’s ascribed antioxidant properties in the gut might be more important than previously considered, thus providing further food for thought for the role of lignin in the human and animal diet.

## Data Availability

Data will be made available on request.

## Supporting Information Available

Chemical composition of wheat bran; lignin content of destarched and deproteinated wheat bran; pyrolysis-GC-MS structural features of wheat bran and lignin fractions; ^31^P NMR lignin characterization; HSQC NMR of dioxane extract (PDF)
