# Supplementary material for: Comprehensive Structural Characterization of Wheat Bran Lignin
Source: J Agric Food Chem. 2025 Apr 5;73(15):9136–43. doi: 10.1021/acs.jafc.4c11880 (PMC12007093; doi:10.1021/acs.jafc.4c11880)
Supplement: Supplementary file 1 — jf4c11880_si_001.pdf [file jf4c11880_si_001.pdf]

Supporting Information for

# Comprehensive structural characterization of wheat bran lignin

Gijs van Erven[a, b*], Romy J. Veersma[a], Mirjam A. Kabel[a]

[a]Wageningen University & Research, Laboratory of Food Chemistry, Bornse Weilanden 9, 6708 WG, Wageningen, The Netherlands.

[b]Wageningen Food and Biobased Research, Bornse Weilanden 9, 6708 WG, Wageningen, The Netherlands

*Corresponding author. Tel: +31 317 487010. E-mail address: gijs.vanerven@wur.nl

Number of pages: 4

Number of tables: 4

Number of figures: 1

**Table S1.** Chemical composition of untreated wheat bran. Average and standard deviation of duplicates. NSP: non-starch polysaccharides, expressed as anhydrosaccharides.

| | Content (% w/w dry matter) |
|---|---|
| **Starch** | 10.5 ± 0.2 |
| **NSP** | 45.3 ± 0.7 |
| **Arabinose** | 9.7 ± 0.4 |
| **Xylose** | 18.7 ± 0.6 |
| **Glucose** | 13.4 ± 0.3 |
| **Uronic acid** | 2.2 ± 0.0 |
| **Protein** | 17.6 ± 0.5 |
| **Ash** | 6.7 ± 0.0 |
| **Fat** | 3.9 ± 0.0 |
| **Lignin (Klason)[a]** | 13.3 ± 0.7 |
| **Esterified ferulic acid** | 0.5 ± 0.0 |

[a]Acid-insoluble lignin (AIL) corrected for ash and protein + acid-soluble lignin (ASL)

**Table S2.** Lignin content of destarched and deproteinated wheat bran. Average and standard deviation of duplicates (Klason), and triplicates (pyrolysis-GC-MS).

| | Content (% w/w dry matter) |
|---|---|
| **AIL corrected for ash, protein and fat** | 5.4 ± 0.4 |
| **ASL** | 1.5 ± 0.1 |
| **AIL+ASL** | 6.9 ± 0.4 |
| **$^{13}$C-IS py-GC-MS 'all products'** | 8.4 ± 0.3 |
| **$^{13}$C-IS py-GC-MS 'excluding H-units'** | 7.9 ± 0.3 |
| **$^{13}$C-IS py-GC-MS 'excluding 4-VG'** | 3.1 ± 0.3 |
| **$^{13}$C-IS py-GC-MS 'excluding H-units and 4-VG'** | 2.6 ± 0.2 |

AIL acid-insoluble lignin; ASL acid-soluble lignin; 4-VG 4-vinylguaiacol

**Table S3.** $^{13}$C-IS py-GC-MS relative abundance of lignin-derived compounds in wheat bran fractions, and in wheat straw used as reference. Corrected for relative response factors (RRF) and relative abundance of $^{13}$C-IS analogues. Average and standard deviation of triplicates.

| | Wheat bran destarched deproteinated | Crude wheat bran lignin | Purified wheat bran lignin | Wheat straw |
|---|---|---|---|---|
| **Lignin subunits (%)** | | | | |
| **H** | 7.2 ± 0.0 | 15.3 ± 0.8 | 11.7 ± 0.4 | 18.4 ± 0.8 |
| **G** | 77.9 ± 0.3 | 52.7 ± 0.6 | 47.7 ± 0.8 | 58.2 ± 1.1 |
| **S** | 14.9 ± 0.3 | 32.0 ± 0.2 | 40.6 ± 1.2 | 23.5 ± 0.2 |
| **S/G** | 0.19 ± 0.0 | 0.61 ± 0.0 | 0.85 ± 0.0 | 0.40 ± 0.0 |
| ***t*-CouA**[a] | 5.8 ± 0.1 | 6.7 ± 0.1 | 5.9 ± 0.2 | 3.8 ± 0.1 |
| ***t*-ConA** | 45.4 ± 0.1 | 39.9 ± 0.1 | 35.4 ± 0.2 | 61.9 ± 0.5 |
| ***t*-SinA** | 48.8 ± 0.1 | 53.4 ± 0.1 | 58.7 ± 0.3 | 34.3 ± 0.5 |
| ***t*-SinA/*t*-ConA** | 1.08 ± 0.0 | 1.34 ± 0.0 | 1.66 ± 0.0 | 0.55 ± 0.0 |
| **Structural moieties (%)** | | | | |
| **Unsubstituted** | 5.9 ± 0.2 | 13.1 ± 0.1 | 11.0 ± 0.7 | 6.6 ± 0.2 |
| **Methyl** | 3.2 ± 0.0 | 5.7 ± 0.1 | 4.9 ± 0.3 | 4.3 ± 0.0 |
| **Vinyl** | 67.8 ± 0.9 | 42.4 ± 0.6 | 31.1 ± 2.4 | 39.2 ± 0.1 |
| **4-VP**[d] | 2.0 ± 0.1 | 5.8 ± 0.8 | 4.0 ± 0.2 | 12.5 ± 0.5 |
| **4-VG**[e] | 63.2 ± 1.0 | 29.5 ± 0.3 | 21.0 ± 1.8 | 23.6 ± 0.7 |
| **$C_\alpha$-ox** | 2.3 ± 0.0 | 4.9 ± 0.0 | 5.3 ± 0.1 | 4.5 ± 0.0 |
| **$C_\beta$-ox**[f] | 0.6 ± 0.0 | 1.8 ± 0.1 | 1.6 ± 0.1 | 1.0 ± 0.0 |
| **$C_\gamma$-ox** | 18.4 ± 1.1 | 28.2 ± 0.6 | 42.1 ± 3.6 | 40.5 ± 0.0 |
| **Miscellaneous** | 1.7 ± 0.0 | 3.8 ± 0.0 | 3.9 ± 0.2 | 3.8 ± 0.1 |
| **PhCγ**[g] | 20.4 ± 1.1 | 33.6 ± 0.5 | 47.6 ± 3.4 | 45.3 ± 0.1 |

[a] *trans*-coumaryl alcohol. [b] *trans*-coniferyl alcohol. [c] *trans*-sinapyl alcohol. [d] 4-vinylphenol. [e] 4-vinylguaiacol. [f] excluding diketones. [g] phenols with intact α,β,γ carbon side chain.

**Table S4.** $^{31}$P NMR phenolic content of wheat bran lignin isolate. Contents per gram sample corrected for Klason lignin content of isolate.

| OH unit | OH content (mmol/g lignin) |
|---|---|
| aliphatic | 2.2 |
| carboxylic acid | 0.8 |
| syringyl + condensed guaiacyl | 0.8 |
| guaiacyl | 0.5 |
| *p*-hydroxyphenyl | 0.3 |
| Total phenolic OH | 1.6 |

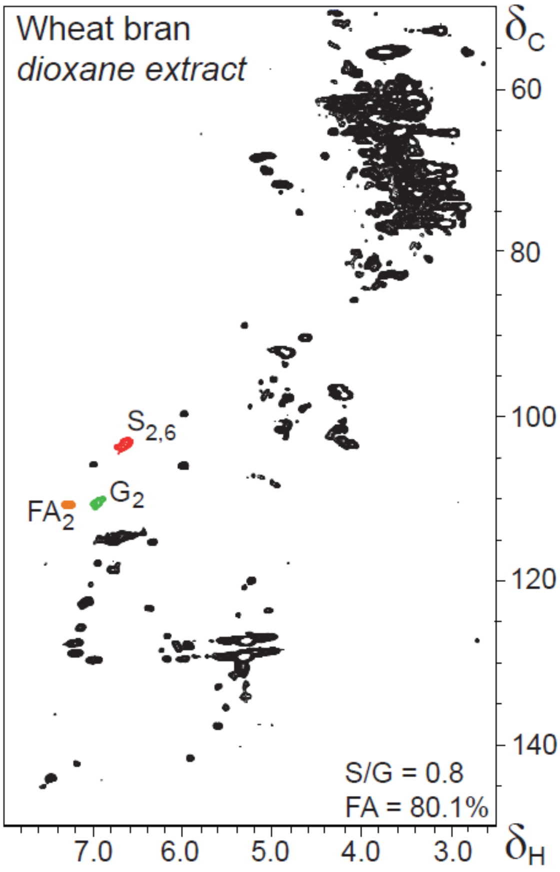


**Figure S1**. HSQC NMR spectrum of wheat bran dioxane extract. $S_{2,6}$; $G_2$ and $FA_2$ signals are colored according to Figure 2; all other signals in black.
